# Cannabis Use: An Uncommon Cause of Hypokalemia-Induced Acute Paralysis

**DOI:** 10.7759/cureus.44393

**Published:** 2023-08-30

**Authors:** Angad Singh, Andre Apostolatos, Ajay Iyer, Benny Escobedo, Melissa Middlemas

**Affiliations:** 1 Internal Medicine, Overland Park Regional Medical Center, Overland Park, USA; 2 Internal Medicine, Kansas City University of Medicine and Biosciences, Kansas City, USA

**Keywords:** acute hypokalemic paralysis, cannabis induced hypokalemia, cannabis use complications, severe hypokalemia etiologies, severe hypokalemia induced paralysis

## Abstract

Severe hypokalemia can have life-threatening complications such as significant muscle weakness, ileus, rhabdomyolysis, and respiratory failure. Here, we report a case of a 33-year-old male who presented with worsening lower extremity weakness and falls after smoking marijuana for six months. Initial labs showed severe hypokalemia. EKG was remarkable for a first-degree AV block, widened QRS complex, and ST segment depression. Intravenous potassium replacement resulted in complete resolution of lower extremity motor weakness. Our case highlights the underdiagnosed association of marijuana use with clinically significant hypokalemia and the rare presentation of severe hypokalemia with acute paralysis.

## Introduction

Hypokalemia is a common electrolyte abnormality encountered in clinical practice. Severe hypokalemia (serum potassium <2.5 mmol/l), can lead to life-threatening complications such as significant muscle weakness, ileus, rhabdomyolysis, and respiratory failure [1]. Cannabis use is an under-recognized cause of hypokalemia. Here, we are presenting a case of acute paralysis due to cannabis-induced severe hypokalemia in a young adult.

## Case presentation

A 33-year-old male with a history of attention-deficit hyperactivity disorder (ADHD) and recently diagnosed hypertension presented to the emergency room due to worsening lower extremity weakness. He had experienced an episode of weakness one day ago after he smoked marijuana, and fell while going to the restroom. He fell again the next day when trying to get out of bed and was brought to the ED by his wife. On detailed questioning, the patient reported that he had been experiencing episodes of weakness almost every other week over the last two months, usually a couple of hours after smoking marijuana. He also reported increased appetite and binge eating the day after smoking marijuana. He reportedly procured the marijuana six months ago from a shop in Colorado and had been smoking this every weekend.

The patient denied any headache, vision or hearing changes, speech difficulty, upper extremity weakness, numbness, vomiting, diarrhea, bowel or bladder incontinence, saddle anesthesia, diuretic use, or drug use besides marijuana. His home medications included a 20 mg racemic mixture of amphetamine and methylphenidate twice daily, which he had been taking for two years without any adverse events, and losartan 50 mg daily.

**Figure 1:**
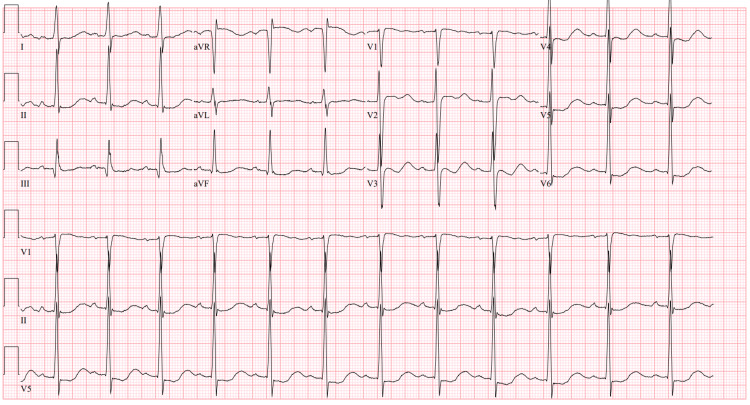
EKG on admission

On admission, patient's blood pressure 145/92 with a heart rate of 83, respiratory rate of 20, temperature 36.8° C and oxygen saturation 99% on room air. His physical examination was significant for symmetrical loss of complete motor function and decreased deep tendon reflexes in bilateral lower extremities without sensory abnormalities. Cranial nerve testing and upper extremity neurological examination were unremarkable. The cardiac and abdominal examination was unremarkable too. Initial labs were consistent with severe hypokalemia (Table 1). Urine toxicology was positive for amphetamines and marijuana. An electrocardiogram (EKG) showed sinus rhythm with first-degree AV block, widened QRS, and ST segment depression in the lateral and inferior leads (Figure 1). Imaging studies including head and lumbar spine CT were negative for any acute abnormalities.

**Table 1:** Initial lab values on hospital admission

Lab test	Result (lab reference range)
WBC	5.7 x 10^3^/uL (4.1-11.1 x 10^3^/uL)
Hb	14 g/dl (13.5-16.5 g/dl)
Plt	225 x 10^3^/uL (126-225 x 10^3^/uL)
Sodium	141 mmol/l (135-146 mmol/L)
Potassium	1.6 mmol/l (3.6-5.2 mmol/l)
Chloride	105 mmol/l (100-108 mmol/l)
Blood urea nitrogen	16 mg/dl (6-22 mg/dl)
Bicarbonate	25 mmol/dl (21-32 mmol/l)
Creatinine	0.8 mg/dl (0.5-1.3 mg/dl)
Random blood glucose	147 mg/dl (70-99 mg/dl)
Magnesium	1.6 mg/dl (1.6-2.3 mg/dl)
Urinary potassium	4 mmol/l
Urinary creatinine	29.3 mg/dl (20-300 mg/dl)
Creatinine kinase	190 units/l (26-308 units/l)

**Figure 2:**
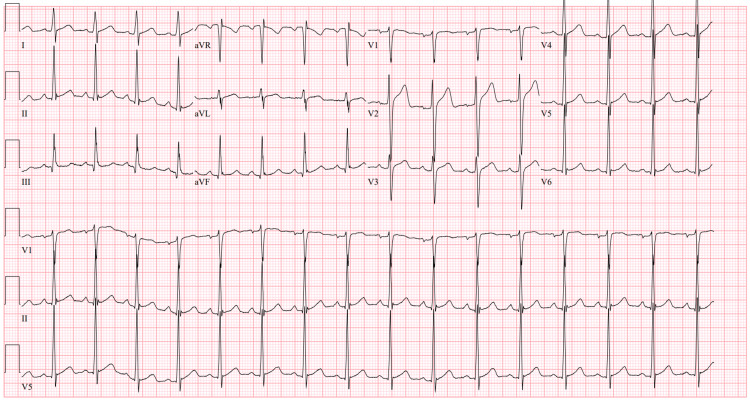
EKG 24 hours later at the time of discharge

The patient was given a total of 120 milliequivalents of IV potassium, which helped correct serum potassium levels to 4.1 over the next 24 hours. A repeat EKG showed a normal sinus rhythm without any significant abnormalities (Figure 2). At the time of discharge, the patient had complete resolution of lower extremity motor weakness with normal deep tendon reflexes.

## Discussion

Cannabis use has gained widespread popularity due to its recreational and medicinal properties. However, emerging evidence suggests that cannabis consumption can lead to rare but severe complications, including hypokalemia. The case reported here highlights this often underdiagnosed association presenting with a rare complication of acute paralysis.

The underlying mechanisms responsible for severe hypokalemia associated with cannabis use remain poorly understood. Cannabis-induced hypokalemia is thought to be due to a transcellular potassium shift. Cannabinoids primarily exert many of their cellular and organ effects by activating the Gi/o protein-coupled, cannabinoid type 1 (CB1) and type 2 (CB2) receptors [2]. CB1 receptor activation has been shown to activate G protein-coupled inwardly rectifying potassium (GIRK) and A-type (fast inactivating) potassium channels through a CB1 receptor-dependent signaling mechanism [3,4]. Galiègue et al. described how both cannabinoid receptors, CB1 and CB2, were expressed in several peripheral tissues [5]. This patient's hyperdynamic response to potassium replacement supports the hypothesis of a potassium shift over potassium wasting or poor intake. This leads us to conclude that the likely etiology of his hypokalemia and subsequent lower extremity muscle paralysis was a transcellular potassium shift.

Initially, the differential for acute weakness and paralysis in our previously healthy 33-year-old male was broad and included Guillain-Barré syndrome and tick paralysis. However, as hypokalemia came to the foreground, the focus shifted to determining its cause. In order to ascertain the etiology of hypokalemia in this case, we considered common differentials such as potassium loss via the gastrointestinal system, urinary potassium wasting, and decreased potassium intake [6]. These proposed causes were felt to be unlikely in this patient because he had no history of diarrhea or vomiting, and his urinary potassium level of 4 mmol/l suggested that hypokalemia was not due to urinary potassium wasting. Additionally, the patient did not report starting any new low-calorie diet, and had normal skin turgor with moist mucous membranes suggesting adequate hydration. He did not have a family history supporting a genetic disorder and did not report any previous episodes in childhood, making hypokalemic periodic paralysis less likely. Additionally, his age at presentation and low urinary potassium excretion essentially ruled out Gitelman syndrome. He had no exercise intolerance or weakness after exertion prior to this episode, and thus, myasthenia gravis and metabolic myopathies did not fit this clinical picture. The patient was also taking a 20 mg racemic mixture of amphetamine and methylphenidate twice daily. Charach et al. found that methylphenidate, a piperidine derivative structurally related to amphetamines, has mild hyperglycemic and hypokalemic effects [7]. However, the mechanisms by which these effects happen as well as whether amphetamines can cause similar clinical findings have not been well studied. The patient's low dose of Adderall is not expected to have caused profound hypokalemia, especially since the patient reported taking the same dose for two years without side effects or electrolyte abnormalities.

In a previous study, Simonsen et al. documented an uncommon instance involving a young man who experienced hypokalemic paresis following the use of recreational cannabis [8]. Another comparable case was reported by Taskiran et al., wherein a young male developed hypokalemia after using synthetic cannabis, but his condition improved quickly upon receiving potassium supplementation [9]. Since our patient had a temporal association of weakness with cannabis use and there were no other probable causes, we propose that this case of hypokalemic paralysis was cannabis-induced.

## Conclusions

Our case report highlights an uncommon but important association between cannabis use and severe hypokalemia-induced acute paralysis. Clinicians should be vigilant in considering this potentially life-threatening complication in patients with unexplained neuromuscular symptoms following cannabis consumption. Understanding the underlying mechanisms and raising awareness about cannabis-induced hypokalemia is crucial for timely recognition and management.

## References

[1] Engle JE (1990). Clinical physiology of acid-base and electrolyte disorders. JAMA.

[2] Lin YF (2021). Potassium channels as molecular targets of endocannabinoids. Channels (Austin).

[3] Pertwee RG (2015). Endocannabinoids and their pharmacological actions. Handbook of Experimental Pharmacology.

[4] Ameri A (1999). The effects of cannabinoids on the brain. Prog Neurobiol.

[5] Galiègue S, Mary S, Marchand J (1995). Expression of central and peripheral cannabinoid receptors in human immune tissues and leukocyte subpopulations. Eur J Biochem.

[6] Unwin RJ, Luft FC, Shirley DG (2011). Pathophysiology and management of hypokalemia: a clinical perspective. Nat Rev Nephrol.

[7] Charach G, Karniel E, Grosskopf I, Rabinovich A, Charach L (2020). Methylphenidate has mild hyperglycemic and hypokalemia effects and increases leukocyte and neutrophil counts. Medicine (Baltimore).

[8] Simonsen SK, Rittig NF, Poulsen PL, Svart MV (2022). Hypokalemic paresis in a 26-year-old man after recreational cannabis use. Am J Case Rep.

[9] Taskiran B, Mutluay R (2014). A case of hypokalemia with synthetic cannabinoid use. Med Sci.

